# Exploring the connection between RNA splicing and intellectual disability

**DOI:** 10.1016/j.gde.2025.102322

**Published:** 2025-02-08

**Authors:** Anthony Caputo, Ashleigh E Schaffer

**Affiliations:** Department of Genetics and Genome Sciences, Case Western Reserve University School of Medicine, Cleveland, Ohio, United States

## Abstract

Intellectual disability (ID) is a broad diagnostic category that encompasses individuals with impaired cognitive ability. While these disorders have heterogeneous causes, recent developments in next-generation sequencing (NGS) are revealing the prevalence of genetic etiologies. In particular, germline mutations in genes that affect RNA splicing are increasingly common causes of ID disorders. Research to elucidate the functional relationship between splicing and neurodevelopment is critical since molecular therapeutics require a nuanced understanding of the pathological mechanism. In this review, we first summarize the trends that have led to the discovery of the RNA splicing–ID relationship, then discuss recent progress and future directions for research surrounding RNA splicing in neurodevelopment. Finally, we speak on how these results may serve as the foundation for burgeoning therapies.

## Introduction

Intellectual disability (ID) comprises a group of neurodevelopmental disorders characterized by impaired cognition and adaptive learning that affects 1–3% of the global population [1,2]. While ID has heterogenous etiologies, recent clinical practices are revealing the overwhelming role of genetics in these disorders [3,4]. There is now up to a 60% chance of a causal genetic mutation being identified in an affected individual [5]. As the repertoire of documented ID-causing mutations grows, we get a clearer understanding of which genes are most important for neurodevelopment and function [6,7]. This information has highlighted RNA splicing as a key pathway perturbed in this process, with splicing-associated mutations frequently resulting in ID [6,8] (Table 1).

RNA splicing is a co-transcriptional process in which noncoding introns of newly transcribed RNA are removed to produce functionally mature RNA. This process is carried out by a group of proteins and RNAs collectively known as the spliceosome [9]. The spliceosome recognizes specific sequence elements within the nascent transcript, then catalyzes the removal of introns and ligation of exons. Splicing is a vital cellular process, as it is required for the functional maturity of nearly all human mRNAs, as well as some noncoding RNAs. For a more thorough discussion of RNA splicing, see this review [10].

Splicing-associated mutations that cause ID can be broadly classified as one of two types: variants in the spliceosome that cause transcriptome-wide processing defects and variations in splicing sequence elements that only affect the mutated transcript. This distinction is important because the type of mutation dictates potential therapeutic avenues. In this review, we first summarize major trends that have led to the drastic increase in identifying causal variants of ID. Then, we discuss recent progress and current gaps in research surrounding the link between splicing mutations and neurodevelopment. Finally, we explore potential therapeutic opportunities for affected individuals.

## Technological advancements accelerate variant identification in intellectual disability

Within the past few years, the number of publications reporting on causal variants of ID has been on the rise. This trend can be largely attributed to two interconnected practices: developments in next-generation sequencing (NGS) as a diagnostic tool and increased data sharing within and between researchers and clinicians.

NGS is a high-throughput method used to determine the sequence of DNA or RNA. While this review focuses on discoveries made primarily by applying NGS to patient genomes, it is important to note that NGS of RNA is also an extraordinarily valuable tool. RNA sequencing can be used to validate genome sequencing, or it can be applied independently to reveal variations in RNA composition that may be highly relevant, particularly to diseases involving RNA splicing [11].

As a clinical diagnostic tool, NGS is commonly used to sequence an individual’s genome to identify mutations that may be responsible for their particular diagnosis. When used in the clinic, the technology is commonly divided into two categories: whole-genome sequencing (WGS) and whole-exome sequencing (WES), which probe either the entire genome or just the protein-coding regions, respectively. Both follow a similar workflow: collection of patient DNA, library preparation, sequencing, bioinformatic analysis, and variant interpretation [12]. NGS has been utilized in clinical practices since 2012 [13], but its utility has significantly increased since then [14]. This is due to myriad factors, such as technological progress that has decreased the cost of implementation [15], efforts to standardize the pipeline for maximal efficiency [16,17], and development and optimization of variant annotation software. Annotation software are bioinformatic algorithms that utilize known DNA–protein relationships to predict the effects of novel mutations on their gene products. They are commonly used to assess whether the disease-associated variant identified in patients is likely to affect protein expression or function using this measure as a proxy for pathogenicity.

While variant annotation is used in both types of NGS, WES has gained substantial popularity recently because it is less expensive and requires simpler bioinformatic analyses than WGS while retaining similar detection rates [18,19]. These advantages also make WES particularly suitable for trio analysis, a technique in which the affected individual and their parents all undergo genome sequencing. This technique is particularly effective at identifying *de novo* causal variants of rare diseases [20]. However, there are disadvantages of WES, namely, its scope of detection. WGS can screen the entire genome, while WES only detects protein-coding regions. The significance of this limitation will be discussed more thoroughly later in this review. Despite this limitation, the aforementioned technological advancements have made NGS, and particularly WES, a practical, cost-effective, and efficient tool for identifying causal mutations in individuals with ID. The fruit of this clinical practice not only informs patient decisions but also produces a vast catalog of ID-associated variants. When available to researchers, these data can be explored to discover meaningful patterns about the biology underlying ID disorders.

**Figure 1:**
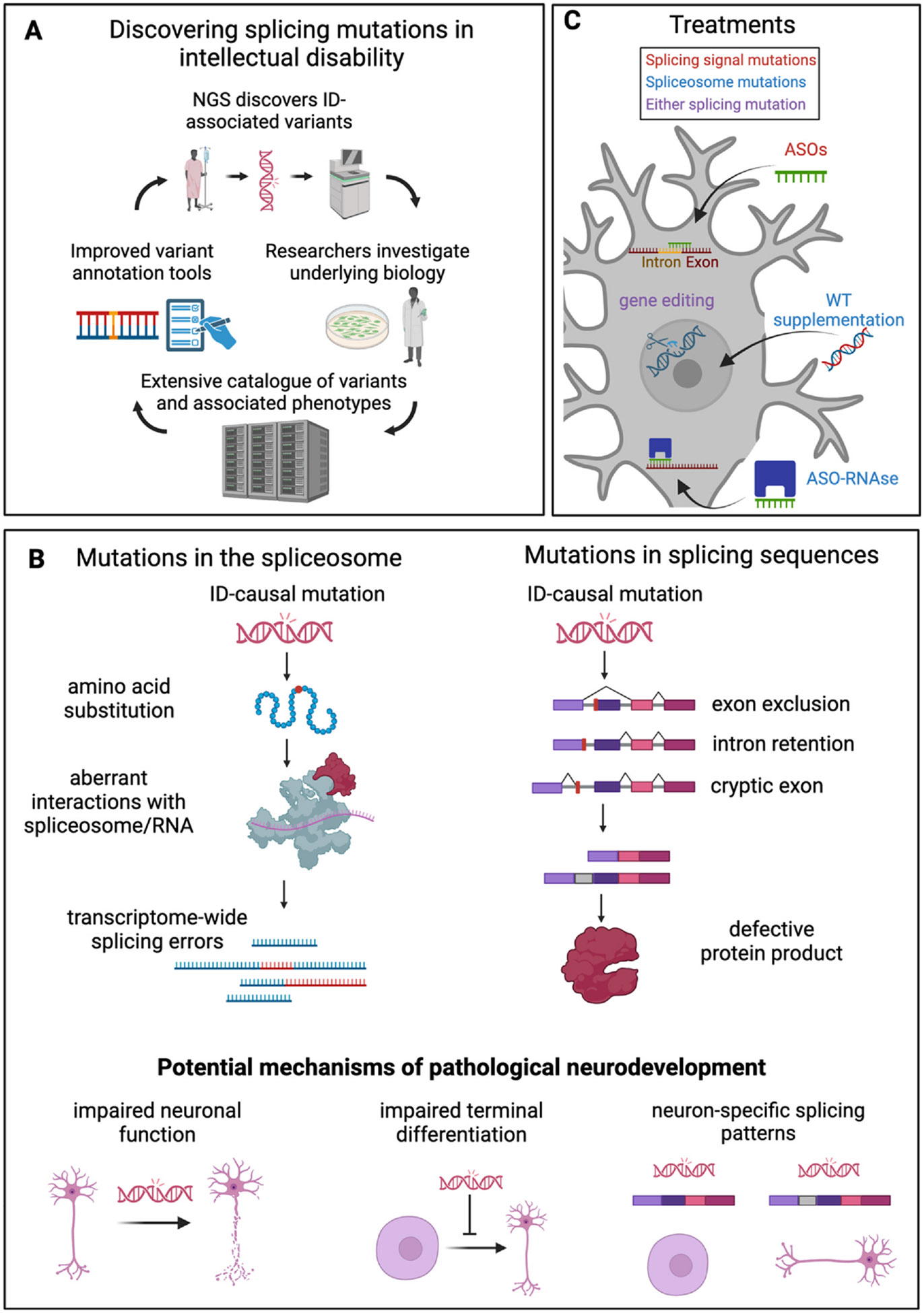
An overview of RNA splicing in intellectual disability. **(a)** Visual representation of the collaboration-fueled positive feedback loop that has led to the discovery of ID-associated RNA-splicing mutations. **(b)** Differences between two types of splicing mutations and potential mechanistic explanations for how they cause ID. **(c)** Burgeoning molecular-based prospective therapies for individuals with ID.

Elucidating the molecular impact of the vast multitude of pathogenic variants is an overwhelming task, but data sharing has proven to be an invaluable strategy in this quest for understanding. Variant databases allow clinicians and researchers to upload and search for mutations that have been associated with a phenotype. Services such as PhenoDB [21], GeneMatcher [22], and VariantMatcher [23] are typically used by clinicians to upload variants of unknown significance that are linked to a particular patient phenotype. Researchers then use this information to identify patterns in which novel variants are recurrently associated with ID disorders. These discoveries then serve as a springboard for investigating the molecular origin of pathogenicity, which, when found, can be deposited into databases such as FAVOR [24], gnomAD [25], and ClinVar [26]. These databases are also variant-phenotype catalogs but with much more extensive functional annotation for each variant. They contain detailed information on how specific mutations alter their gene products to affect cellular function. This nuanced understanding also feeds back into improving variant annotation software, creating a positive feedback loop in which the more we learn about how variants affect phenotype, the better we get at predicting the effects of novel variation. As a testament to this, the value of both types of databases is well recognized [27-30], with many publications utilizing these tools to identify novel disease genes and determine how they cause pathology. In the context of ID, an ever-growing number of these genes are in some way related to the process of RNA splicing (Figure 1a).

## Mutations in the spliceosome

RNA splicing is performed by the spliceosome, a dynamic complex of small nuclear RNAs (snRNAs) and over 200 interacting proteins [9]. Nearly all human genes contain introns, so splicing is necessary in all cell types. However, diagnostic WES has revealed a reproducible pattern in which mutations in RNA-binding proteins (RBPs) of the spliceosome frequently result in pathological neurodevelopment (Figure 1b). Why and how mutations in ubiquitously expressed proteins produce a tissue-specific phenotype is still not totally understood.

Recent efforts to understand the mechanistic origins of ID have provided some possible explanations for the neuro-specific effects of spliceosome mutations. RNA-seq was used to assess changes in mRNA splicing caused by mutations in HNRNPC [31], WBP4 [32], and RBM42 [33]. In each case, mutations caused transcriptome-wide alternative splicing (AS), but AS occurred most frequently in genes involved in neural function and neurodevelopment. These studies converge on a relatively simple explanation in which RBPs regulate the splicing of only a subset of transcripts, and mutations in RBPs that splice neuronally expressed transcripts thereby cause neurological disorders. In line with this hypothesis, ID-causal variants in NAB2 [34] and PRMT9 [35] lead to widespread splicing defects concentrated in transcripts implicated in neuronal function. In these cases, the molecular mechanisms are slightly upstream, as neither NAB2 nor PRMT9 are spliceosome proteins. Rather, NAB2 plays a role in mRNA methylation, a modification known to influence splicing patterns [36,37], and PRMT9 functionally alters a splicing RBP, SF3B2, through methylation [35]. While these studies offer simple and elegant explanations for the neuro-specific impact of spliceosome variants, other work has shown that there is undoubtedly more to the story.

FMR1 variants cause transcriptome-wide aberrant splicing, and three genes necessary for neuronal function are alternatively spliced [38]. In this example, it is understandable how the mutation would impair neurodevelopment. However, neuronal genes were not disproportionately represented in the AS analysis, so in this case, it is not clear why other organ systems are not also significantly impacted. This result argues that not all of ID can be explained by spliceosome RBPs displaying selectivity for their downstream targets.

There are several important events that occur during tissue and organ system development, such as cellular proliferation and migration, formation of extracellular matrices, cell–cell interactions, and cellular differentiation. Cellular differentiation is the process by which unspecialized and immature cells transition into more specialized states. Interestingly, variants in MLPKIP that cause ID also produce unbiased, transcriptome-wide splicing aberrations that are associated with impaired keratinocyte differentiation [39]. This lends support to an alternative hypothesis wherein generalized pathological splicing preferentially impacts highly specialized cell types due to the overwhelming importance of splicing during cellular differentiation [40-42]. Since neurons are a complex, highly specialized, and terminally differentiated cell type of the nervous system, this organ system is particularly compromised by splicing perturbances. In support of this hypothesis, many individuals with ID display comorbidities of the heart [43] and skin [44]; tissues also composed primarily of terminally differentiated cells.

With support for both hypotheses, and the strong possibility that both are at least partially valid, it is still not entirely clear why neurons are particularly vulnerable to spliceosome mutations. However, every year more publications are revealing novel variants in spliceosome proteins as causative for ID [45-47]. To understand how a spliceosome mutation causes ID, it is incredibly helpful to first understand the physiological role of the spliceosome protein in question. Recently, one investigation aimed to mechanistically characterize the role of different splicing factors [48]. This group individually knocked down over 300 genes encoding spliceosome components or splicing RBPs in HeLa cells and performed RNA-seq to assess AS. The authors discovered ‘splicing factor networks’, groups of splicing proteins that have similar effects on AS to one another. This intriguing mechanistic investigation helps to elucidate the complicated and historically enigmatic properties of the spliceosome. The results of this study should prove beneficial to future researchers attempting to understand the link between the spliceosome and ID.

Future mechanistic studies that seek to understand the effects of ID-causal mutations on splicing patterns are sure to be informative, especially if they take into consideration two variables not well accounted for in current investigations. First is the use of physiologically relevant model systems. Most current splicing analyses utilize mRNA collected from patient blood. While we can still glean important information from these data, we may obtain a more nuanced understanding by modeling mutations in the cell types that are most relevant to the disorder. With the growing practicality of induced pluripotent stem cells, it seems reasonable for future studies to compare the effects of patient mutations on splicing patterns between otherwise isogenic cell types. Such investigations would help to nail down the neuro-specific phenotypes caused by these mutations. Another worthy consideration is the effect of noncoding RNAs on splicing. WES is the current method of choice for identifying ID mutations, but an inherent limitation of this methodology is its inability to detect regulatory RNAs, such as snRNAs, that make up a significant portion of the spliceosome. To emphasize this point, a recent analysis of WGS data from over 5000 individuals with ID and 45 000 unaffected controls found mutations in the gene encoding U4 snRNA to be the most common cause of ID [49]. Therefore, efforts to understand the role of regulatory RNAs in ID are sure to be fruitful. Still, unveiling the precise role of the spliceosome in ID would provide insight into only a subset of these disorders, as many cases are linked to mutations that only affect splicing of a single transcript.

## Mutations in splicing sequence elements

Splicing sequence elements are motifs within newly transcribed RNAs that are recognized by RBPs of the spliceosome. Two types of sequence elements, splice sites and auxiliary elements, collectively dictate proper intron–exon recognition. Splice site sequences span the intron–exon border and delineate this boundary. Fidelity to a consensus sequence offers a high probability of proper splicing, but deviations from the consensus are common. Therefore, splice site recognition often needs to be modulated by auxiliary sequences located farther away. Mutations in either type of splicing sequence element can lead to disrupted splicing, which manifests as intron retention, exon exclusion, or cryptic exon inclusion. Any type of splicing aberration will likely disrupt proper expression and function of the protein product, serving as a potential mechanism for pathogenicity. As such, these types of mutations are often identified as causing ID (Figure 1b).

Many splice site variants have been identified using a combination of WES and splicing prediction technologies [50,51]. Detecting an intronic sequence with an exon-targeting methodology may seem counterintuitive, but technical aspects of WES allow for capture of intronic sequences within 10 base pairs of exons. This gives WES the ability to identify splice site mutations at annotated intron–exon boundaries. In some cases, there is a reasonable connection between the function of the gene that contains the mutant splice site and pathological neurodevelopment. Such is the case for splice site mutations in *SYNGAP1* [52], which encodes a protein canonically involved in synaptic plasticity, and *PLXNB2* [53], which has a well-known role in axonal guidance. In other situations, the connection is less clear. *ATRX, TRIP12, AP1S2*, and *AGO1* encode proteins involved in chromatin remodeling, protein degradation, endocytosis, and small RNA-mediated gene silencing pathways, respectively. Despite these cellular processes being ubiquitous, splice site mutations in these genes still lead to brain-specific phenotypes, such as ID [54-57].

Future work on splicing sequence elements and ID should address a few current gaps in the field. First is the mechanistic link between ID-associated splicing sequence variants in ubiquitous genes and a neurodevelopmental phenotype. In these cases, it remains unclear whether these mutations produce the same splicing patterns in all cell types. To answer this question, many groups are utilizing long-read RNA-seq [58], a technology capable of sequencing a full-length RNA transcript in a single read. This allows for the detection of complex alternative splicing patterns and splicing kinetics, a difficult feat with short-read RNA-seq technology [58]. Using long-read RNA-seq, several groups have revealed brain region-specific patterns of AS under physiological conditions [59,60]. Not only are splicing patterns different between brain regions, but they also differ across development and differentiation [61]. Therefore, it is possible that splicing sequence mutations only result in mis-spliced transcripts in neurons, or even certain subtypes of neurons, while other cell types are less affected. Continuous use of long-read RNA-seq in the context of disease-relevant mutations in isogenic cell types will surely provide further insight into this matter.

The second mystery is the extent to which cryptic splicing is implicated in ID. Currently, WES is the method-of-choice to screen for mutations in splicing sequences because it is cost-effective and can easily probe the most common splice sites. However, there is evidence of mutations in auxiliary splicing sequences in deep intronic regions leading to cryptic exon inclusion and ID [62]. This type of mutation cannot be detected by WES and therefore relies on WGS for elucidation. It is highly likely that many more examples like this exist, yet to be discovered because of an underutilization of WGS. An additional consideration that can be investigated with either NGS methodology is the role of exonic splicing sequence elements in ID. Single-nucleotide variants in the coding sequence often cause missense or synonymous mutations. In many cases, these are interpreted to be nonpathogenic by variant annotation due to their negligible effect on protein structure. However, these types of mutations can also influence splicing patterns [63]. Therefore, when interpreting the potential pathogenicity of exonic variants, careful attention should be paid not only to how mutations affect amino acid sequence, but also how they can affect splicing patterns. This type of analysis will likely lead to the discovery of pathogenic variants that may provide useful insight into underlying biology of ID disorders.

## Prospective therapeutics

Current treatment options for individuals with ID are typically limited to behavioral intervention and specialized education [64,65]. Arguably, the most effective therapies for patients with a genetic etiology would be those that target the molecular origin of the disorder (Figure 1c). To do this, a case-by-case understanding of each unique causal variant is necessary. Foundations such as the National Organization for Rare Disorders [66] and N-lorem [67] are geared toward just that, developing clinical therapeutics for extremely rare genetic disorders. In many cases of ID, a prerequisite for developing this kind of personalized medicine is understanding the molecular consequences of causal variants both in the spliceosome and in splicing signals.

Both types of splicing mutations are instances of monogenic pathology. In this way, both can be corrected by gene editing technologies that replace the mutant allele with the wild-type counterpart. In fact, there is an ongoing clinical trial that is using CRISPR/Cas9 to try to correct missense mutations in *MECP2* that cause ID [68]. While MECP2 is a chromatin-binding protein, there is no reason why the same technology could not be applied to splicing mutations. However, this approach suffers from non-negligible disadvantages such as limited cell permeability of reagents and low editing efficiency [69]. While there are some U.S. Food and Drug Administration (FDA)-approved gene editing strategies, they treat blood disorders and can therefore utilize *ex vivo* gene editing to increase efficacy. *In vivo* gene editing is still in its infancy, and for this reason, alternative strategies are being pursued for ID disorders.

According to gnomAD [25], many of the identified spliceosome variants underlying ID are predicted to be loss-of-function. In these cases, supplementation of the normal reference (WT) protein product in the presence of the mutant gene is sufficient to treat the disease. This is simpler than gene editing, which requires both removal of the mutant allele and replacement with the WT allele. WT supplementation only requires that the WT copy be placed somewhere in the genome where it can be transcribed. As a testament to the practicality of the supplementation approach, multiple ongoing clinical trials aim to treat ID by delivering WT gene copies of *ABCD1* [70], *MECP2* [71], and *IDS* [72] to individuals harboring homozygous loss-of-function mutations in these genes.

Mutations in splicing signals are amenable to an additional therapeutic option not available to loss-of-function variants in the spliceosome. Antisense oligonucleotides (ASOs) are single-stranded synthetic DNA or RNA that bind to mRNA via base pair complementarity. They are commonly designed to target splicing sequence elements, blocking their interactions with RBPs to restore physiological splicing patterns. *In vitro* studies have shown the promise of ASOs in combating mutations in splicing sequences that cause ID. Trinucleotide repeat expansions in *FMR1* disrupt protein function via intron retention that can be reverted with ASOs targeting the cryptic splice site [38]. In another example, splice site mutations in *SYNGAP1*, a gene important for synaptic plasticity, also result in intron retention that disrupts the function of the protein product. ASOs targeting nearby auxiliary signals were successfully used to correct splicing and revert the neurodegenerative phenotype in a physiologically relevant induced pluripotent stem cell-derived model of neuronal differentiation [52]. Importantly, ASOs also show promise in the clinic. The first ASO therapy was approved by the FDA in 2016 to treat spinal muscular atrophy, and 10 more have been approved since then [73]. In addition to their capacity for correcting splicing mutations, there are rare instances of dominant-negative or gain-of-function missense mutations in the spliceosome that could be treatable with ASOs.

While most spliceosome variants that cause ID are loss-of-function, there is evidence of dominant-negative protein products as well. In one example, heterozygous dominant-negative variants in *TRA2B* were identified as causal for ID [74]. In cases like these, the capacity of the mutant gene product to disrupt wild-type interactions necessitates that the mutant allele be eliminated, and therefore, WT supplementation will not be effective. However, specialized ASOs that recruit RNAses to degrade a target mRNA [75] rather than alter mRNA processing may be effective in these scenarios. There are a few FDA-approved strategies that successfully utilize this mechanism of action, such as Formivirsen, Inotersen, Mipomersen, and Volanosorsen. In theory, these could be leveraged as therapeutics that eradicate dominant-negative or gain-of-function spliceosome mutations that cause ID.

## Conclusions

Since ID is an umbrella term for a complex suite of neurological phenotypes affecting cognitive ability, there is no single mechanism of etiology. This makes unraveling the nuanced molecular origins of pathology across all affected individuals a daunting task. However, recent advances in genetic diagnostic tools and increased collaborative efforts among researchers and clinicians are proving to be invaluable practices in accelerating ID research. These trends have revealed genetic perturbations of RNA splicing to be prominently and recurrently associated with ID. While initial mechanistic studies have led to some hypotheses connecting splicing and ID, we are a long way off from a unified consensus, if one exists. To investigate the relationship between neurodevelopment and splicing, future work will need to understand how splicing mutations preferentially affect the nervous system. Tools such as long-read RNA sequencing and models of isogenic cell types harboring patient mutations are sure to provide insight into this enigmatic relationship. These investigations are necessary for the treatment of ID since molecular therapeutics require an individualized understanding of the genetic underpinnings of the disorder. Currently, gene editing and ASOs seem to offer the most promise for these conditions, but rapid technological and conceptual advances are creating an exciting and unpredictable future for the field.

## Figures and Tables

**Table 1:** Hypothesized mechanisms of ID-associated splicing mutations.

Hypothesized pathological mechanism	Genes with [reference]
Spliceosome mutation causes AS of genes important for neuronal function	HNRNPC [31], WBP4 [32], RBM42 [33], SON [46]
Spliceosome mutation impairs cellular differentiation across different lineages	MLPKIP [39]
Spliceosome mutation with unknown mechanism	FMR1 [38], SRSF1 [45], SRRM2 [47], U4 snRNA [49]
Splicing sequence element mutation causes mis-splicing of transcript important for neuronal function	SYNGAP1 [52], PLXNB2 [53], DLG4 [62]
Splicing sequence element mutation in ubiquitously expressed gene, unknown mechanism of neuropathology	ATRX [54], TRIP12 [55], AP1S2 [56], AGO1 [57]

## Data Availability

No data were used for the research described in the article.
